# Access to Specialty Care for Commercially Insured Youths With Type 1 and Type 2 Diabetes

**DOI:** 10.1001/jamanetworkopen.2024.5656

**Published:** 2024-04-05

**Authors:** Christine A. March, Amy R. Byerly, Linda Siminerio, Elizabeth Miller, Scott Rothenberger, Ingrid Libman

**Affiliations:** 1 Department of Pediatrics, University of Pittsburgh School of Medicine, Pittsburgh, Pennsylvania; 2 Pediatric Endocrinology, UPMC Children’s Hospital of Pittsburgh, Pittsburgh, Pennsylvania; 3 Department of Medicine, University of Pittsburgh School of Medicine, Pittsburgh, Pennsylvania; 4 Adolescent and Young Adult Medicine, UPMC Children’s Hospital of Pittsburgh, Pittsburgh, Pennsylvania; 5 Center for Research on Health Care Data Center, University of Pittsburgh School of Medicine, Pittsburgh, Pennsylvania

## Abstract

**Importance:**

Youths with type 2 diabetes are at higher risk for complications compared with peers with type 1 diabetes, though few studies have evaluated differences in access to specialty care.

**Objective:**

To compare claims with diabetes specialists for youths with type 1 vs type 2 diabetes and the association between specialist claims with multidisciplinary and acute care utilization.

**Design, Setting, and Participants:**

This cross-sectional study used Optum Clinformatics Data Mart commercial claims. Individuals included in the study were youths younger than 19 years with type 1 or 2 diabetes as determined by a validated algorithm and prescription claims. Data were collected for youths with at least 80% enrollment in a commercial health plan from December 1, 2018, to December 31, 2019. Statistical analysis was performed from September 2022 to January 2024.

**Main Outcomes and Measures:**

The primary outcome was the number of ambulatory claims from an endocrine and/or diabetes physician or advanced practice clinician associated with a diabetes diagnosis code; secondary outcomes included multidisciplinary and acute care claims.

**Results:**

Claims were analyzed for 4772 youths (mean [SD] age, 13.6 [3.7] years; 4300 [90.1%] type 1 diabetes; 472 [9.9%] type 2 diabetes; 2465 [51.7%] male; 128 [2.7%] Asian, 303 [6.4] Black or African American, 429 [9.0%] Hispanic or Latino, 3366 [70.5%] non-Hispanic White, and 546 [11.4%] unknown race and ethnicity). Specialist claims were lower in type 2 compared with type 1 diabetes (incidence rate ratio [IRR], 0.61 [95% CI, 0.52-0.72]; *P* < .001) in propensity score–weighted analyses. The presence of a comorbidity was associated with increased specialist claims for type 1 diabetes (IRR, 1.07 [95% CI, 1.03-1.10]) and decreased claims for type 2 diabetes (IRR, 0.77 [95% CI, 0.67-0.87]). Pooling diagnosis groups and adjusted for covariates, each additional specialist claim was associated with increased odds of a claim with a diabetes care and education specialist (odds ratio [OR], 1.31 [95% CI, 1.25-1.36]), dietitian (OR, 1.14 [95% CI, 1.09-1.19]), and behavioral health clinician (OR, 1.16 [95% CI, 1.12-1.20]). For acute care claims, each additional specialist claim was associated with increased odds of admission (OR, 1.17 [95% CI, 1.11-1.24]) but not for emergency claims (OR, 1.03 [95% CI, 0.98-1.82]).

**Conclusions and Relevance:**

This cross-sectional study found that youths with type 2 diabetes were significantly less likely to have specialist claims despite insurance coverage, indicating other barriers to care, which may include medical complexity. Access to diabetes specialists influences engagement with multidisciplinary services. The association between increasing ambulatory clinician services and admissions suggests high utilization by a subgroup of patients at greater risk for poor outcomes.

## Key Points

**Question:**

How does access to specialty care differ between youths with type 1 and type 2 diabetes?

**Findings:**

In this cross-sectional study of commercial claims for 4772 youths, those with type 2 diabetes had approximately 40% fewer claims with a diabetes specialist compared with peers with type 1 diabetes, a significant difference.

**Meaning:**

These results suggest that youths with type 2 diabetes were less likely to have specialty care despite insurance coverage, indicating other barriers to care.

## Teaser

This cross-sectional study compares claims with diabetes specialists for youths with type 1 vs type 2 diabetes and the association between specialist claims with multidisciplinary and acute care utilization.

## Introduction

Following diagnosis, the goals of ongoing pediatric type 1 and 2 diabetes care are to optimize glucose control, prevent complications, and promote well-being. Both national and international guidelines recommend that youths with diabetes be treated by a multidisciplinary care team encompassing diabetes education, nutrition, and psychosocial support.^1,2^ This care includes visits every 3 months with a pediatrician or advanced practice clinician (APC, which includes nurse practitioner and physician assistant) specializing in diabetes to evaluate management, growth and development, education needs, and risk for comorbidities.^2^ Visits should include annual assessments in partnership with multidisciplinary team members, including a diabetes care and education specialist (DCES), dietitian, psychologist, and social worker.^2^

Though comprehensive, some families may encounter challenges in trying to engage with the health care system this frequently. Barriers to care may be at the patient level or diabetes center level and include access to adequate health insurance, availability of and distance to specialists, medical and appointment-related costs, or lack of a consistent medical home.^3^ For youths with type 1 diabetes, the SEARCH for Diabetes in Youth study^4^ found that more than 80% of families reported at least 1 barrier, most often costs, poor continuity, and communication challenges. Barriers related to costs and lack of a regular clinician were more prevalent among families with public insurance and/or low income.^4^

The effect of these barriers on specialty care delivery has been evaluated to some extent in pediatric type 1 diabetes. Two studies examining data from a tertiary care hospital each found that approximately 15% of youths missed 2 or more appointments per year with diabetes clinicians.^5,6^ Missed appointments were more common among those on premixed insulin and with indicators of socioeconomic disparities.^6,7^ Having fewer appointments than recommended has been correlated to adverse outcomes in some studies, including higher hemoglobin A_1c_^5,6,7^ and increased risk for diabetic ketoacidosis (DKA).^6^ Though these studies signal the importance of specialty medical follow-up for youths with type 1 diabetes, they are limited by substantial heterogeneity in design, use older datasets predating modern treatments, and only represent data from large academic centers in urban settings.

Data are more limited for type 2 diabetes. Two small studies found that 42% to 60% of youths were lost to follow-up within 2 years after diagnosis.^8,9^ Though youths with type 2 diabetes may be at higher risk for acute care utilization, the association between outpatient specialty follow-up and health outcomes has been inadequately studied in this population. As youths with type 2 diabetes are at higher risk for long-term microvascular and macrovascular complications compared with their peers with type 1 diabetes,^10^ understanding current access to specialty diabetes follow-up is important to design interventions that engage these youths.

To understand access to specialty care in a national sample, the objective of this study was to compare patterns of specialty ambulatory care services for children with type 1 or type 2 diabetes using a commercial claims-based dataset. Using commercial claims enabled us to reduce the known barriers of health care access and medical costs when assessing for engagement with specialty medical services. A secondary objective was to explore the association between the frequency of diabetes specialist claims with multidisciplinary care access and acute care utilization. We hypothesized that youths with type 2 diabetes would be less likely to have specialty care and more likely to require acute care utilization.

## Methods

This cross-sectional study analyzed national claims data for youths with type 1 or type 2 diabetes in the United States. As this study involved an analysis of deidentified claims data, it was considered exempt by the University of Pittsburgh institutional review board and did not require informed consent. This study followed the Strengthening the Reporting of Observational Studies in Epidemiology (STROBE) reporting guideline.

We used Optum’s deidentified Clinformatics Data Mart (CDM) Database, derived from commercial insurance claims in the United States. The CDM is statistically deidentified under the Expert Determination method consistent with the Health Insurance Portability and Accountability Act and managed according to CDM customer data use agreements. CDM administrative claims submitted for payment by clinicians and pharmacies are verified, adjudicated, and deidentified prior to inclusion. These data, including patient-level enrollment information, are derived from claims submitted for all medical and pharmacy health care services with information related to health care costs and resource utilization. The population is geographically diverse, spanning all 50 states.

The cohort included youths younger than 19 years with type 1 or type 2 diabetes and at least 80% insurance enrollment between December 1, 2018, and December 31, 2019. Insurance enrollment was defined as the proportion of the date range when the participant had coverage with United Health Care. Diabetes type was determined by a validated algorithm using the ratio of *International Classification of Diseases, Ninth Revision (ICD-9)* and* ICD-10* codes supplemented by prescription claims.^11^ The algorithm’s sensitivity and specificity are greater than 90%; the addition of prescription claims increases the positive predictive value.^11^ The diagnostic and procedural codes used to classify individuals, outcomes, and covariates are included in eTable 1 in Supplement 1. Cohort selection is diagrammed in eFigure 1 in Supplement 1.

The primary outcome was number of ambulatory claims in our date range from an endocrine and/or diabetes physician or APC associated with a diabetes diagnosis code. Participants with more than 12 ambulatory claims in the date range were excluded, as these were felt to be unlikely to all represent true clinical encounters. Secondary outcomes were categorized as follows: claims with DCES, dietician, or behavioral health clinicians (yes or no) and acute care claims, defined as at least 1 emergency claim, at least 1 admission, and at least 1 DKA.

Covariates included gender, census region (eTable 2 in Supplement 1), household income, diabetes duration, insulin pump and continuous glucose monitor (CGM) use, and medical complexity defined by the Pediatric Complex Chronic Condition Classification (CCC) Version 2, which classifies comorbidities by diagnosis code along 12 categories.^12^ Race and ethnicity were reported descriptively but excluded from analyses, as these were imputed in the CDM and may not accurately represent the identities of the included participants. Duration of diabetes was estimated by the difference between the first available date with an *ICD-9* or *ICD-10* diabetes diagnosis code, extending back to 2010, and our date range. Device use was determined by *ICD-10* codes.

### Statistical Analysis

Baseline patient characteristics, as well as primary and secondary outcomes, were first summarized descriptively and compared by diagnosis using *t* tests and χ^2^ tests. To compare the rate of clinician claims by diagnosis, we conducted propensity score–weighted Poisson regression to minimize confounding in relation to patient characteristics. Stabilized inverse probability weights were estimated using logistic regression as a function of age, gender, diabetes duration, device use, region, income, and comorbidity. Participants with weights greater than 4 (99.5 percentile) were trimmed to reduce bias from overweighting individual observations. For each diagnosis, we then assessed for associations between clinical and sociodemographic variables with the primary outcome using a univariable Poisson regression. Next, multivariable models were fit with significant variables at the *P* < .20 level to adjust for possible confounding. Lastly, we assessed for interactions with diagnosis to identify meaningful differences in the association with the primary outcome by diagnosis. All Poisson regression models were offset by person-years of enrollment.

Propensity score–weighted logistic regression was used to examine differences in secondary outcomes by diagnosis. Evaluation of these outcomes used univariable and multivariable logistic regression modeling. As the number of claims for secondary outcomes was low, models assessing the association between the number of diabetes clinician claims with secondary outcomes included participants with both diagnoses except for DKA, which was fit for type 1 diabetes only. For multidisciplinary claims, the models were adjusted for sociodemographic variables only; for acute care claims, the models were adjusted for all covariates and behavioral health care. All analyses were completed from September 2022 to January 2024 using Stata version 17.0 (StataCorp) with significance determined by a 2-sided *P* < .05.

## Results

Among 4772 participants included in the analytic cohort, 4300 (90.1%) had type 1 diabetes; 472 (9.9%) had type 2 diabetes; 2465 (51.7%) were male; mean (SD) age was 13.6 (3.7) years; mean (SD) duration of diabetes was 2.4 (2.5) years; 128 (2.7%) were Asian, 303 (6.4%) were Black or African American, 429 (9.0%) were Hispanic or Latino, 3366 (70.5%) were non-Hispanic White, and 546 (11.4%) had unknown race and ethnicity. Descriptive characteristics for the sample and by diagnosis are included in Table 1. Participants with type 2 diabetes were more likely to have at least 1 comorbidity and to have diagnoses in multiple CCC categories, suggesting higher medical complexity than individuals with type 1 diabetes.

**Table 1.** Descriptive Data of the Analytic Cohort, Overall and by Diagnosis

Characteristic	No. (%)			*P* value
	Overall (N = 4772)	Type 1 diabetes (n = 4300)	Type 2 diabetes (n = 472)	
Age, mean (SD), y	13.6 (3.7)	13.4 (3.8)	15.4 (2.6)	<.001
Gender				
Male	2465 (51.7)	2293 (53.3)	172 (36.4)	<.001
Female	2307 (48.3)	2007 (46.7)	300 (63.6)	
Race and ethnicity				
Asian	128 (2.7)	103 (2.4)	25 (5.3)	<.001
Black or African American	303 (6.4)	233 (5.4)	70 (14.8)	
Hispanic or Latino	429 (9.0)	333 (7.8)	96 (20.3)	
Non-Hispanic White	3366 (70.5)	3137 (72.7)	239 (50.6)	
Unknown	546 (11.4)	504 (11.7)	42 (8.9)	
Duration of diabetes, mean (SD), y	2.4 (2.5)	2.5 (2.5)	1.3 (1.6)	<.001
Insulin pump use	NA^a^	2715 (63.1)	NA^a^	NA
CGM use	2614 (54.8)	2591 (60.3)	23 (4.9)	<.001
Region^b^				
East North Central	779 (16.3)	714 (16.6)	65 (13.8)	<.001
East South Central	224 (4.7)	199 (4.6)	25 (5.3)	
Middle Atlantic	237 (5.0)	214 (5.0)	23 (4.9)	
Mountain	634 (13.3)	579 (13.5)	55 (11.6)	
New England	130 (2.7)	121 (2.8)	9 (1.9)	
Pacific	430 (9.0)	400 (9.3)	30 (6.4)	
South Atlantic	1010 (21.2)	896 (20.8)	114 (24.1)	
West North Central	626 (13.1)	583 (13.6)	43 (9.1)	
West South Central	688 (14.4)	581 (13.5)	107 (22.7)	
Household income, $				
<$40 000	395 (8.3)	322 (7.5)	73 (15.5)	<.001
$40 000-49 000	169 (3.5)	136 (3.2)	33 (7.0)	
$50 000-59 000	188 (4.0)	153 (3.6)	35 (7.4)	
$60 000-74 000	290 (6.1)	250 (5.8)	40 (8.5)	
$75 000-99 000	550 (11.5)	486 (11.3)	64 (13.5)	
≥$100 000	2176 (45.6)	2044 (47.5)	132 (28.0)	
Unknown	1004 (21.0)	909 (21.1)	95 (20.1)	
At least 1 CCC comorbidity	1444 (30.3)	1138 (26.5)	306 (64.8)	<.001
No. of categories if at least 1 CCC comorbidity, mean (SD)	1.4 (0.8)	1.3 (0.7)	1.7 (1.0)	<.001

Abbreviations: CCC, Complex Chronic Condition Classification Version 2, which defines diagnosis codes in 12 categories; CGM, continuous glucose monitor; NA, not applicable.

^a^ Fewer than 2% of participants with type 2 diabetes used an insulin pump.

^b^ See eTable 2 in Supplement 1 for details about which states are included in the regions as defined by the Clinformatics Data Mart Database.

Participants with type 1 diabetes had significantly more mean (SD) claims with a diabetes clinician than those with type 2 diabetes (4.0 [1.8] vs 2.0 [2.3] claims; *P* < .001). Fewer than one-quarter of participants (23.3%) with type 2 diabetes had 4 or more clinician claims, compared with 60% of participants with type 1 diabetes (Table 2; eFigure 2 in Supplement 1). In propensity score–weighted analyses, youths with type 2 diabetes had a significantly lower rate of clinician claims compared with youths with type 1 diabetes per person-year (IRR, 0.61 [95% CI, 0.52-0.72]).

**Table 2.** Diabetes Clinician, Multidisciplinary, and Acute Care Claims, Overall and by Diagnosis

Claim	No. (%)			*P* value	Adjusted^a^	
	Overall (N = 4772)	Type 1 diabetes (n = 4300)	Type 2 diabetes (n = 472)		OR (95% CI)	*P* value
Diabetes clinician claims, No.						
0	238 (5.0)	69 (1.6)	169 (35.8)	<.001	IRR, 0.61 (95% CI, 0.52-0.72)	<.001
1	296 (6.2)	224 (5.2)	72 (15.3)			
2	546 (11.4)	474 (11.0)	72 (15.3)			
3	1001 (21.0)	952 (22.1)	49 (10.4)			
4	1202 (25.2)	1166 (27.1)	36 (7.6)			
≥5	1489 (31.2)	1415 (32.9)	74 (15.7)			
Multidisciplinary claims						
DCES	662 (13.9)	638 (14.8)	24 (5.1)	<.001	0.58 (0.28-1.20)	.14
Dietitian	560 (11.7)	507 (11.8)	53 (11.2)	.72	1.18 (0.70-1.99)	.53
Behavioral health	1120 (23.5)	978 (22.7)	142 (30.1)	<.001	1.36 (0.99-1.88)	.06
Acute care claims						
Emergency	326 (6.8)	289 (6.7)	38 (7.8)	.36	1.16 (0.66-2.05)	.60
Admission	315 (6.6)	280 (6.5)	35 (7.4)	.45	1.00 (0.53-1.86)	.99
DKA	NA	184 (4.3)	NA	NA	NA	NA

Abbreviations: DCES, diabetes care and education specialist; DKA, diabetic ketoacidosis; IRR, incidence rate ratio; NA, not applicable; OR, odds ratio.

^a^ The adjusted IRR and OR are presented for type 2 diabetes with type 1 diabetes as the reference. Models are adjusted for background characteristics using inverse probability weighting with stabilized weights incorporating the unadjusted prevalence of type 1 and 2 diabetes in the sample.

In multivariable Poisson regression analyses, younger age (IRR, 0.99 [95% CI, 0.98-0.99]), shorter duration (IRR, 0.99 [95% CI, 0.98-0.99]), CGM use (IRR, 1.09 [95% CI, 1.05-1.12]), and presence of a comorbidity (IRR, 1.07 [95% CI, 1.03-1.10]) were associated with an increasing number of clinician claims for youths with type 1 diabetes per person-year (Table 3). For type 2 diabetes, younger age (IRR, 0.95 [95% CI, 0.93-0.97]), CGM use (IRR, 1.89 [95% CI, 1.51-2.38]), and absence of a comorbidity (IRR, 0.77 [95% CI, 0.67-0.87]) were associated with an increasing number of clinician claims per person-year (Table 4). Results from the interaction-based modeling found that younger age and CGM use had a greater association with increasing clinician claims for youths with type 2 diabetes (*P* = .001 for both). Also in line with the stratified results, we found a significant inverse association between the presence of comorbidity and clinician claims by diagnosis (*P* < .001). For both diagnoses, clinician claims differed significantly regionally, though there was no association with household income as an indicator of socioeconomic status.

**Table 3.** Factors Associated With Diabetes Clinician Visits for Participants With Type 1 Diabetes

Factor	Unadjusted IRR (95% CI)	*P* value^a^	Adjusted IRR (95% CI)	*P* value
Age, y	0.986 (0.982-0.990)	<.001	0.988 (0.984-0.991)	<.001
Gender				
Male	1 [Reference]	NA	1 [Reference]	NA
Female	1.044 (1.013-1.075)	.005	1.043 (1.012-1.075)	.006
Duration of diabetes, y	0.989 (0.984-0.995)	.001	0.989 (0.982-0.995)	.001
Insulin pump use	1.028 (0.996-1.060)	.08	1.018 (0.983-1.053)	.31
CGM use	1.093 (1.060-1.127)	<.001	1.087 (1.051-1.124)	<.001
Region		<.001		<.001
East North Central	1 [Reference]	NA	1 [Reference]	NA
East South Central	0.928 (0.857-1.003)	.06	0.928 (0.857-1.004)	.06
Middle Atlantic	1.047 (0.973-1.126)	.22	1.037 (0.964-1.116)	.33
Mountain	0.891 (0.843-0.941)	<.001	0.881 (0.833-0.931)	<.001
New England	1.009 (0.919-1.108)	.85	1.004 (0.915-1.103)	.93
Pacific	0.931 (0.876-0.990)	.02	0.918 (0.864-0.976)	.006
South Atlantic	0.958 (0.913-1.005)	.08	0.958 (0.913-1.006)	.09
West North Central	0.911 (0.862-0.962)	.001	0.909 (0.860-0.960)	.001
West South Central	0.928 (0.878-0.979)	.007	0.919 (0.870-0.971)	.002
Household income, $		.28		
<$40 000	1 [Reference]	NA	NA	NA
$40 000-49 000	0.941 (0.848-1.044)	.25	NA	NA
$50 000-59 000	1.015 (0.921-1.119)	.76	NA	NA
$60 000-74 000	1.012 (0.931-1.100)	.78	NA	NA
$75 000-99 000	1.036 (0.965-1.113)	.32	NA	NA
≥$100 000	1.037 (0.977-1.101)	.23	NA	NA
Any CCC category	1.049 (1.015-1.085)	.005	1.066 (1.030-1.103)	<.001

Abbreviations: CCC, Complex Chronic Condition Classification Version 2, which defines diagnosis codes in 12 categories; CGM, continuous glucose monitor; IRR, incidence rate ratio; NA, not applicable.

^a^ Covariates significant at the *P* < .20 level in unadjusted analyses were included in the multivariable model. For covariates with multiple subcategories (eg, region, household income), the overall *P* value was used to determine significance for inclusion in the adjusted model. Please see eTable 2 in Supplement 1 for details about which states are included in the regions as defined by the Clinformatics Data Mart Database.

**Table 4.** Factors Associated With Diabetes Clinician Visits for Participants With Type 2 Diabetes

Factor	Unadjusted IRR (95% CI)	*P* value^a^	Adjusted IRR (95% CI)	*P* value
Age, y	0.937 (0.917-0.957)	<.001	0.950 (0.928-0.973)	<.001
Sex				
Male	1 [Reference]	NA	NA	NA
Female	0.981 (0.861-1.119)	.78	NA	NA
Duration of diabetes, years	0.966 (0.927-1.007)	.106	0.964 (0.925-1.005)	.081
Insulin pump use	1.225 (0.802-1.869)	.35	NA	NA
CGM use	2.092 (1.691-2.588)	<.001	1.893 (1.505-2.380)	<.001
Region		.002		.01
East North Central	1 [Reference]	NA	1 [Reference]	NA
East South Central	0.756 (0.542-1.053)	.10	0.751 (0.536-1.051)	.10
Middle Atlantic	0.904 (0.656-1.246)	.54	0.881 (0.635-1.223)	.45
Mountain	0.915 (0.721-1.161)	.46	0.942 (0.740-1.198)	.63
New England	1.401 (0.948-2.071)	.09	1.392 (0.937-2.068)	.10
Pacific	1.024 (0.775-1.352)	.87	1.023 (0.771-1.356)	.88
South Atlantic	0.893 (0.0.730-1.093)	.27	0.863 (0.704-1.058)	.16
West North Central	0.568 (0.420-0.768)	<.001	0.602 (0.445-0.816)	.001
West South Central	0.766 (0.620-0.946)	.01	0.788 (0.636-0.976)	.03
Household income, $		.17		.09
<$40 000	1 [Reference]	NA	1 [Reference]	NA
$40 000-49 000	0.656 (0.477-0.903)	.01	0.668 (0.485-0.920)	.01
$50 000-59 000	0.852 (0.641-1.132)	.27	0.907 (0.681-1.207)	.50
$60 000-74 000	0.913 (0.700-1.190)	.50	0.836 (0.639-1.094)	.19
$75 000-99 000	0.854 (0.676-1.078)	.18	0.886 (0.698-1.124)	.32
≥$100 000	0.979 (0.808-1.185)	.83	0.966 (0.793-1.176)	.73
Any CCC category	0.755 (0.664-0.857)	<.001	0.765 (0.671-0.873)	<.001

Abbreviations: IRR, incidence rate ratio; CCC, Complex Chronic Condition Classification Version 2, which defines diagnosis codes in 12 categories; NA, not applicable.

^a^ Covariates significant at the *P* < .20 level in unadjusted analyses were included in the multivariable model. For covariates with multiple subcategories (eg, region, household income), the overall *P* value was used to determine significance for inclusion in the adjusted model. Please see eTable 2 in Supplement 1 for details about which states are included in the regions as defined by the Clinformatics Data Mart Database.

There were 662 DCES claims present (13.9%), 560 dietitian claims (11.7%), and 1120 behavioral health claims (23.5%) (Table 2). Propensity score–weighted analyses revealed no significant difference in the odds of DCES (OR, 0.58 [95% CI, 0.28-1.20]), dietitian (OR, 1.18 [95% CI, 0.70-1.99]), or behavioral claims (OR, 1.36 [95% CI, 0.99-1.88]) for youths with type 2 compared with type 1 diabetes. Pooled for both diagnoses and adjusted for covariates, each additional clinician claim was associated with increased odds of a claim with a DCES by 31% (OR, 1.31 [95% CI, 1.25-1.36]), dietitian by 14% (OR, 1.14 [95% CI, 1.09-1.19]), and behavioral health by 16% (OR, 1.16 [95% CI, 1.12-1.20]) (Figure A).

**Figure.**
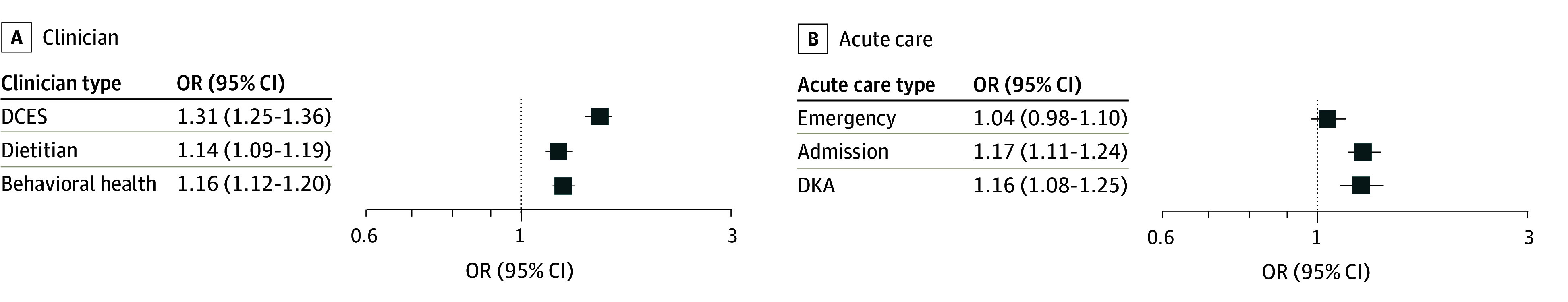
Diabetes Specialist Claims and Claims With Multidisciplinary Clinicians Adjusted for Sociodemographic Variables and Claims for Acute Care Adjusted for Sociodemographic Variables, Device Use, and Mental Health Care Figure shows the association of increasing diabetes specialist claims and claims with multidisciplinary clinicians adjusted for sociodemographic variables (A) and claims for acute care adjusted for sociodemographic variables, device use, and mental health care (B). DCES indicates diabetes care and education specialist; DKA, diabetic ketoacidosis.

Acute claims were present in less than 10% of participants; 326 (6.8%) had an emergency claim (range, 2-30), and 315 (6.6%) had an admission (range, 1-9) (Table 2). The propensity score–weighted model did not identify any difference in the odds of an emergency claim (OR, 1.16 [95% CI, 0.66-2.05]) or admission (OR, 1.00 [95% CI, 0.53-1.86]) by diagnosis. Though not associated with emergency claims, each additional clinician claim was associated with increased odds of admissions by 17% (OR, 1.17 [95% CI, 1.11-1.24]) and DKA by 16% (OR, 1.16 [95% CI, 1.08-1.25]) (Figure B). Further examination found a bimodal distribution by the number of clinician claims. Admissions were more common among those with 0 to 1 (7.1%) or 5 or more (9.5%) clinician claims compared with 2 to 4 claims (range, 4.6%-5.4%; *P* < .001). Similarly, DKA was more common in participants with 0 to 1 (4.8%) or 5 or more (6.0%) claims compared with 2 to 4 claims (range, 3.0%-3.6%; *P* = .004).

## Discussion

In a large dataset of commercially insured children, youths with type 2 diabetes were less likely to have ambulatory claims with a diabetes specialist compared with peers with type 1 diabetes. The proportion who saw a specialist at least every 3 months in accordance with recognized guidelines^1,2^ was 3 times higher for participants with type 1 diabetes. More than half of participants with type 2 diabetes had 0 to 1 claims during the date range. This supports prior reports that a high proportion of youths with type 2 diabetes are lost to specialty follow-up within a few years of diagnosis.^8,9^ Infrequent diabetes specialist visits represent missed opportunities to adjust treatment in a population at high risk for glycemic failure and complications.^10^

The discrepancy in diabetes clinician claims between youths with type 1 and type 2 diabetes in our study is suggestive of persistent barriers to care outside of insurance coverage. One such barrier may be having a usual source of care, an important predictor of meeting medical needs for children^13^ and adults with type 2 diabetes.^14^ Distance to a specialist may also influence routine appointment attendance, though prior studies have yielded conflicting findings.^15,16^ We found that US census region was significantly associated with specialist claims. Though we cannot examine narrower geographic boundaries in this data, the observed variability indicates regional differences in care delivery and may suggest disparities in available pediatric subspecialty diabetes care. If access to a specialist is impractical, it is possible that youths with type 2 diabetes may rely on primary care for ongoing management.^17^ However, to our knowledge, there are no data to substantiate that youths with type 2 diabetes are more often managed by primary care clinicians. Indeed, SEARCH for Diabetes in Youth reported that similar proportions of youths with type 1 and 2 diabetes (>80%) identified pediatric endocrinology as their medical home.^18^ Though our study did not capture primary care claims, other studies have suggested that reliance on primary care is not common until young adulthood.^19^ Future research is needed to assess whether youths with type 2 diabetes are receiving necessary diabetes management elsewhere.

Another potential barrier relates to medical complexity. Although having a comorbidity was associated with increased rate of clinician claims for youths with type 1 diabetes, the opposite was seen for type 2, a statistically significant difference. Youths with type 2 diabetes were more than twice as likely to have another diagnosis within the pediatric CCC, and they were more likely to have a diagnosis in a nonmetabolic (endocrine) category, including neurologic, cardiovascular, and gastrointestinal. This may place greater strain on families to see multiple specialists and may lead to children not receiving the full range of needed health services.^20^ Various strategies to support families, including patient navigation, parent and/or peer coaches, and community health workers, may be useful and cost-effective in improving clinic follow-up to address related health outcomes, though the data remain limited.^21^

Diabetes specialist claims were significantly associated with multidisciplinary claims, suggesting that having fewer visits limits access to other services. Patients may be likely to see multidisciplinary clinicians with their diabetes specialist concurrently at routine appointments. However, this should be interpreted with caution, as the number of multidisciplinary claims in our sample was low. It is unclear if youths are not receiving services or if claims are not filed. Centers may not bill for services if there is low reimbursement.^22^ In contrast, a 2016 survey of 93 primarily academic pediatric endocrine practices reported that, among all patients with diabetes, 86.8% had a visit with a DCES, 71.3% with a dietitian, and 46.4% with a behavioral health specialist.^23^ The small number of private practices reported higher DCES-to-patient and dietitian-to-patient ratios, which likely affects the ability to routinely offer these services.^23^

Though prior studies indicate that youths with type 2 diabetes have higher acute care utilization,^24,25^ we did not observe differences by diagnosis. This discrepancy may relate to data source, as those studies relied on clinical registries rather than commercial claims and included non-US populations with different health care systems. This suggests that access and social factors, rather than diagnosis, may be more influential in acute care use. Interestingly, more clinician claims were associated with higher odds for admissions and DKA, contrary to the literature.^6^ This was driven by participants with more than the recommended number of ambulatory claims who may be seen more frequently because they are at higher risk for complications for other reasons. Indeed, a small number of participants had multiple claims. This suggests that a higher frequency of care does not necessarily translate to improved outcomes, and other approaches may be more beneficial to optimize diabetes management in these patients. Our findings reinforce the need for continued investigation into how to appropriately tailor care to address health needs and prevent both underutilization and overutilization in different populations.

### Limitations

As with all claims-based research, our study has limitations. First, our population with type 2 diabetes differs demographically from other reports.^26^ This may relate to data source, as race and socioeconomic variables are imputed. For this reason, we chose to exclude race and ethnicity from our analyses, which limited our ability to explore well-described inequities related to race and ethnicity for either diagnosis. Second, publicly insured patients are not included in the CDM. Many youths with type 2 diabetes rely on Medicaid in the US,^20^ which may affect the generalizability. Another limitation relates to the classification by diabetes type by billing diagnosis codes; however, a strength of this study includes the use of a validated algorithm to identify cases, which we made more stringent by using pharmaceutical claims. This contributed to several exclusions, for example, due to low continuous enrollment in the health plan associated with the CDM. Diagnosis date was estimated based upon first available diabetes claim; it is possible some participants experienced more frequent visits if recently diagnosed. As the dataset includes claims only, we are likely missing unbilled multidisciplinary encounters, which may be informative in better understanding the distribution of these services nationwide. Claims data do not include other health indicators, such as hemoglobin A_1c_. As this study was cross-sectional and included youths only, we did not assess for diabetes complications. Longitudinal data would better assess associations between ambulatory care in childhood and the risk for future complications.

## Conclusions

To our knowledge, this study is among the first studies to compare patterns in pediatric specialty diabetes care using a national, commercial claims–based dataset. Although 60% of youths with type 1 diabetes had 4 claims with a diabetes clinician per year, this metric was met by fewer than one-quarter of participants with type 2 diabetes. Claims were most significantly affected by age, device use, region, and presence of a medical comorbidity. Though clinician claims were significantly associated with claims from multidisciplinary clinicians, the overall low number of multidisciplinary claims suggests challenges with access and billing. Lastly, the association between clinician claims and acute care utilization is complex; other factors that are unmeasured may be contributing to barriers to care and increased demands for acute care in select participants.

## Notes

Supplement 1. **eTable 1.** Diagnosis and Procedural Codes used for Diabetes Classification, Outcomes, and Covariates **eTable 2.** State Classifications for US Census Regions **eFigure 1.** Flow Diagram of Subject Exclusions for Final Analytic Cohort Selection **eFigure 2.** Percentages of Youth With Increasing Numbers of Diabetes Clinician Claims by Diagnosis

Supplement 2.
Data Sharing Statement

## References

[1] ElSayed NA, Aleppo G, Aroda VR, ; on behalf of the American Diabetes Association. 14. Children and adolescents: standards of care in diabetes-2023. Diabetes Care. 2023;46(suppl 1):S230-S253. doi:10.2337/dc23-S014 36507640 PMC9810473

[2] Limbert C, Tinti D, Malik F, . ISPAD Clinical Practice Consensus Guidelines 2022: The delivery of ambulatory diabetes care to children and adolescents with diabetes. Pediatr Diabetes. 2022;23(8):1243-1269. doi:10.1111/pedi.13417 36537530

[3] Hill-Briggs F, Adler NE, Berkowitz SA, . Social determinants of health and diabetes: a scientific review. Diabetes Care. 2020;44(1):258-279. doi:10.2337/dci20-0053 33139407 PMC7783927

[4] Valenzuela JM, Seid M, Waitzfelder B, ; SEARCH for Diabetes in Youth Study Group. Prevalence of and disparities in barriers to care experienced by youth with type 1 diabetes. J Pediatr. 2014;164(6):1369-75.e1. doi:10.1016/j.jpeds.2014.01.035 24582008 PMC4035445

[5] Markowitz JT, Volkening LK, Laffel LM. Care utilization in a pediatric diabetes clinic: cancellations, parental attendance, and mental health appointments. J Pediatr. 2014;164(6):1384-1389. doi:10.1016/j.jpeds.2014.01.045 24612905 PMC4035443

[6] Fortin K, Pries E, Kwon S. Missed medical appointments and disease control in children with type 1 diabetes. J Pediatr Health Care. 2016;30(4):381-389. doi:10.1016/j.pedhc.2015.09.012 26559135

[7] Kaufman FR, Halvorson M, Carpenter S. Association between diabetes control and visits to a multidisciplinary pediatric diabetes clinic. Pediatrics. 1999;103(5 Pt 1):948-951. doi:10.1542/peds.103.5.948 10224170

[8] Reinehr T, Schober E, Roth CL, Wiegand S, Holl R; DPV-Wiss Study Group. Type 2 diabetes in children and adolescents in a 2-year follow-up: insufficient adherence to diabetes centers. Horm Res. 2008;69(2):107-113. doi:10.1159/000111814 18059091

[9] Pulgarón ER, Hernandez J, Dehaan H, . Clinic attendance and health outcomes of youth with type 2 diabetes mellitus. Int J Adolesc Med Health. 2015;27(3):271-274. doi:10.1515/ijamh-2014-0021 25153557

[10] Bjornstad P, Drews KL, Caprio S, ; TODAY Study Group. Long-term complications in youth-onset type 2 diabetes. N Engl J Med. 2021;385(5):416-426. doi:10.1056/NEJMoa2100165 34320286 PMC8697255

[11] Zhong VW, Pfaff ER, Beavers DP, ; Search for Diabetes in Youth Study Group. Use of administrative and electronic health record data for development of automated algorithms for childhood diabetes case ascertainment and type classification: the SEARCH for Diabetes in Youth Study. Pediatr Diabetes. 2014;15(8):573-584. doi:10.1111/pedi.12152 24913103 PMC4229415

[12] Feudtner C, Feinstein JA, Zhong W, Hall M, Dai D. Pediatric complex chronic conditions classification system version 2: updated for ICD-10 and complex medical technology dependence and transplantation. BMC Pediatr. 2014;14:199. doi:10.1186/1471-2431-14-199 25102958 PMC4134331

[13] Devoe JE, Tillotson CJ, Wallace LS, Lesko SE, Angier H. The effects of health insurance and a usual source of care on a child’s receipt of health care. J Pediatr Health Care. 2012;26(5):e25-e35. doi:10.1016/j.pedhc.2011.01.003 22920780 PMC3512198

[14] DeVoe JE, Tillotson CJ, Wallace LS. Usual source of care as a health insurance substitute for U.S. adults with diabetes? Diabetes Care. 2009;32(6):983-989. doi:10.2337/dc09-0025 19252167 PMC2681031

[15] Liese AD, Ma X, Reid L, . Health care access and glycemic control in youth and young adults with type 1 and type 2 diabetes in South Carolina. Pediatr Diabetes. 2019;20(3):321-329. doi:10.1111/pedi.12822 30666775 PMC6456401

[16] Stumetz KS, Yi-Frazier JP, Mitrovich C, Briggs Early K. Quality of care in rural youth with type 1 diabetes: a cross-sectional pilot assessment. BMJ Open Diabetes Res Care. 2016;4(1):e000300. doi:10.1136/bmjdrc-2016-000300 27933188 PMC5129075

[17] Shoemaker A, Cheng P, Gal RL, ; for the Pediatric Diabetes Consortium. Predictors of loss to follow-up among children with type 2 diabetes. Horm Res Paediatr. 2017;87(6):377-384. doi:10.1159/000475595 28505610

[18] Waitzfelder B, Pihoker C, Klingensmith G, ; SEARCH for Diabetes in Youth Study Group. Adherence to guidelines for youths with diabetes mellitus. Pediatrics. 2011;128(3):531-538. doi:10.1542/peds.2010-3641 21859914 PMC3164090

[19] Garvey KC, Finkelstein JA, Zhang F, LeCates R, Laffel L, Wharam JF. Health care utilization trends across the transition period in a national cohort of adolescents and young adults with type 1 diabetes. Diabetes Care. 2022;45(11):2509-2517. doi:10.2337/dc22-0152 36001755 PMC9679267

[20] Silver EJ, Stein RE. Access to care, unmet health needs, and poverty status among children with and without chronic conditions. Ambul Pediatr. 2001;1(6):314-320. doi:10.1367/1539-4409(2001)001<0314:ATCUHN>2.0.CO;2 11888421

[21] Raphael JL, Rueda A, Lion KC, Giordano TP. The role of lay health workers in pediatric chronic disease: a systematic review. Acad Pediatr. 2013;13(5):408-420. doi:10.1016/j.acap.2013.04.015 24011745 PMC3802546

[22] Vigersky RA, Fitzner K, Levinson J; Diabetes Working Group. Barriers and potential solutions to providing optimal guideline-driven care to patients with diabetes in the U.S. Diabetes Care. 2013;36(11):3843-3849. doi:10.2337/dc13-0680 24159181 PMC3816882

[23] Guttmann-Bauman I, Thornton P, Adhikari S, . Pediatric endocrine society survey of diabetes practices in the United States: what is the current state? Pediatr Diabetes. 2018;19(5):859-865. doi:10.1111/pedi.12677 29582520

[24] Auzanneau M, Rosenbauer J, Icks A, . Hospitalization in pediatric diabetes: a nationwide analysis of all admission causes for germany in 2015. Exp Clin Endocrinol Diabetes. 2020;128(9):615-623. doi:10.1055/a-0972-1060 31426109

[25] Sellers EAC, McLeod L, Prior HJ, Dragan R, Wicklow BA, Ruth C. Hospitalization and comorbidity in children with type 2 diabetes. Pediatr Diabetes. 2022;23(6):660-667. doi:10.1111/pedi.13369 35643934

[26] TODAY Study Group. Health care coverage and glycemic control in young adults with youth-onset type 2 diabetes: results from the TODAY2 study. Diabetes Care. 2020;43(10):2469-2477. doi:10.2337/dc20-0760 32778555 PMC7510035

