# Supplementary material for: Access to Specialty Care for Commercially Insured Youths With Type 1 and Type 2 Diabetes
Source: JAMA Netw Open. 2024 Apr 5;7(4):e245656. doi: 10.1001/jamanetworkopen.2024.5656 (PMC10998152; doi:10.1001/jamanetworkopen.2024.5656)
Supplement: Supplement 1. — eTable 1. Diagnosis and Procedural Codes used for Diabetes Classification, Outcomes, and Covariates eTable 2. State Classifications for US Census Regions eFigure 1. Flow Diagram of Subject Exclusions for Final Analytic Cohort Selection eFigure 2. Percentages of Youth With Increasing Numbers of Diabetes Clinician Claims by Diagnosis [file jamanetwopen-e245656-s001.pdf]

Supplemental Online Content

March CA, Byerly AR, Siminerio L, Miller E, Rothenberger S, Libman I. Access to specialty care for commercially insured youths with type 1 and type 2 diabetes. *JAMA Netw Open.* 2024;7(4):e245656. doi:10.1001/jamanetworkopen.2024.5656

eTable 1. Diagnosis and Procedural Codes used for Diabetes Classification, Outcomes, and Covariates

eTable 2. State Classifications for US Census Regions

eFigure 1. Flow Diagram of Subject Exclusions for Final Analytic Cohort Selection

eFigure 2. Percentages of Youth With Increasing Numbers of Diabetes Clinician Claims by Diagnosis

This supplemental material has been provided by the authors to give readers additional information about their work.

eTable 1: Diagnosis and Procedural Codes used for Diabetes Classification, Outcomes, and Covariates

Category	Codes
Diagnosis codes to classify diabetes	Type 1 diabetes: E10.1-E10.9<br>Type 2 diabetes: E11.1-E11.9
Diagnosis codes for diabetic ketoacidosis	E10.10, E10.11, E11.10, E11.11, E13.10, E13.11
Codes excluded from the Pediatric Complex Chronic Condition Classification Version 2	*Metabolic*: Z794, 79641, 74681, V6546, V5391<br>*Transplant*: V4585, V5391<br>*Technology Dependent*: All insulin pump or CGM codes listed below were excluded
Diagnosis codes for insulin pump:	ICD-10: Z9641, V4585, Z4681, V6545, V5391<br>NDC codes: A4222, A4230, A4231, A4232, A4225, E0784, A9274, S9145, S9353, A4224, J1817, K0601, K0602, K0603, K0604, K0604, K0605, S1034
Diagnosis codes for continuous glucose monitors:	CPT codes: 95249, 95250, 95251<br>NDC Codes: A9267, A9277, A9278, K0553, K0554, S1030, S1031, S1035, S1036, S1037
Procedural codes to identify ambulatory encounters with providers	CPT codes 99243, 99244, 99245, 99213, 99214, 99215, 99203, 99204, 99205 if associated with an E10 or E11 ICD-10 code
Procedural codes to identify ambulatory encounters with diabetes care and education specialists	CPT 98960, 98961, 98962; HCPCS G0108, G0109, S9445, S9446
Procedural codes to identify ambulatory encounters with dietitians	CPT 97802, 97803, 97804; HCPCS G0270, G0271, S9470, S9452
Procedural codes to identify ambulatory encounters with behavioral health	CPT 90791, 90832, 90834, 90837, 90839, 90845, 90847, 90849, 90853, 90863

© 2024 March CA et al. *JAMA Network Open.*

eTable 2: State classifications for US census regions

Region	States Included
East North Central	IL, IN, MI, OH, WI
East South Central	AL, KY, MS, TN
Middle Atlantic	NJ, NY, PA
Mountain	AZ, CO, ID, MT, NV, NM, UT, WY
New England	CT, ME, MA, NH, RI, VT
Pacific	AK, CA, HI, OR, WA
South Atlantic	DE, DC, FL, GA, MD, NC, SC, VA, WV
West North Central	IA, KS, MN, MO, NE, ND, SD
West South Central	AR, LA, OK, TX

© 2024 March CA et al. *JAMA Network Open.*

eFigure 1: Flow diagram of subject exclusions for final analytic cohort selection.

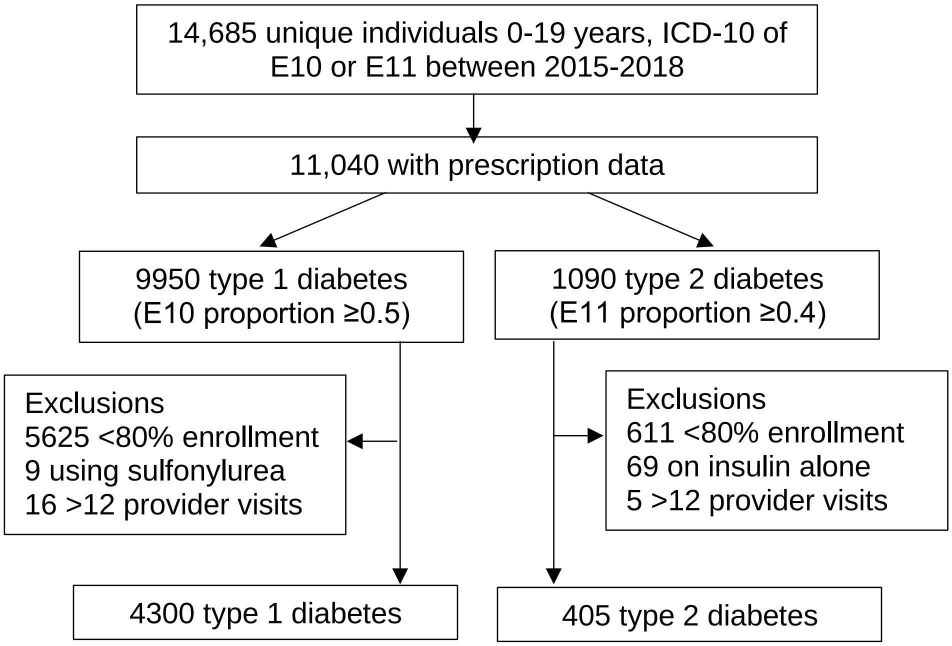

Figure Legend: Validated algorithms were used to identify diagnosis type and supplemented with prescription data. Greater than 80% continuous enrollment was required to maximize the number of encounters associated with claims. We excluded subjects with type 1 diabetes taking a sulfonylurea, as this was likely indicative of monogenic diabetes. We excluded subjects with type 2 diabetes on insulin alone, as these subjects displayed an in-between phenotype between type 1 and 2 diabetes, and their correct classification could not be assured. Subjects with over 12 visits in the calendar year were excluded as this was likely capturing other types of encounters.

© 2024 March CA et al. *JAMA Network Open.*

eFigure 2: Percentages of youth with increasing numbers of diabetes clinician claims by diagnosis.

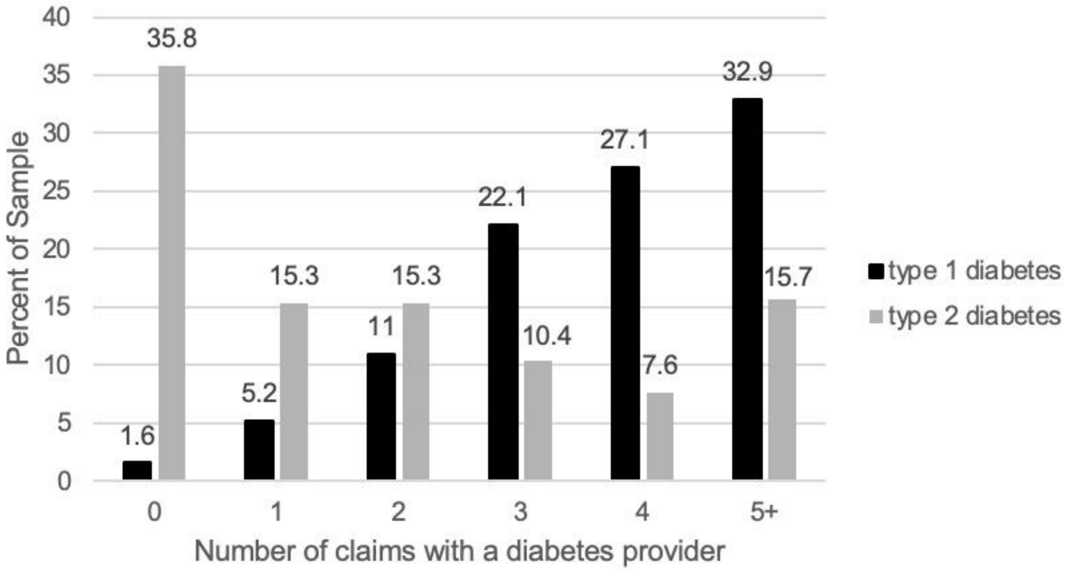

© 2024 March CA et al. *JAMA Network Open.*
