# Supplementary material for: Access to Specialty Care for Commercially Insured Youths With Type 1 and Type 2 Diabetes
Source: JAMA Netw Open. 2024 Apr 5;7(4):e245656. doi: 10.1001/jamanetworkopen.2024.5656 (PMC10998152; doi:10.1001/jamanetworkopen.2024.5656)
Supplement: Supplement 2. — Data Sharing Statement [file jamanetwopen-e245656-s002.pdf]

Data Sharing Statement

March. Access to Specialty Care for Commercially Insured Youths With Type 1 and Type 2 Diabetes. *JAMA Netw Open*. Published April 05, 2024.
doi:10.1001/jamanetworkopen.2024.5656

Data
Data available: No

Additional Information
Explanation for why data not available: The data license agreement between Optum and our institution has changed. We will not have ongoing access to the source data.
